# Supplementary material for: The effects of prophylactic use of paracetamol on body temperature and blood pressure in elderly patients with acute stroke: Data from the PRECIOUS trial
Source: PLoS One. 2026 Feb 25;21(2):e0342937. doi: 10.1371/journal.pone.0342937 (PMC12935189; doi:10.1371/journal.pone.0342937)
Supplement: S1 Table — (DOCX) [file pone.0342937.s001.docx]

**Supplemental Table 2.** Mean systolic and diastolic blood pressure in patients with and without paracetamol

| **Time** | **Systolic blood pressure** | | | | | | | |
| --- | --- | --- | --- | --- | --- | --- | --- | --- |
|  | **No paracetamol** | |  | **Paracetamol** | |  | | |
|  | **Mean (SD)** | **N** |  | **Mean (SD)** | **N** | *DIM (95% CI)* | *Adjusted mean difference^#^* | *p-value* |
| 0 | 153.9 (25.5) | 723 |  | 151.7 (26.5) | 690 | -2.3 (-5.0 – 0.5) | - | - |
| 12h | 145.8 (22.6) | 693 |  | 144.1 (24.1) | 649 | -1.7 (-4.3 – 0.8) | 0.9 (-2.2 – 4.0) | 0.59 |
| 24h | 143.1 (21.1) | 674 |  | 143.2 (23.7) | 632 | 0.1 (-2.3 – 2.5) | 2.1 (-1.0 – 5.2) | 0.19 |
| 36h | 143.5 (20.5) | 644 |  | 144.3 (22.1) | 602 | 0.8 (-1.6 – 3.2) | 2.4 (-0.5 – 5.4) | 0.11 |
| 48h | 142.6 (21.0) | 620 |  | 142.7 (21.5) | 587 | 0.2 (-2.2 – 2.6) | 1.8 (-1.3 – 4.9) | 0.25 |
| 60h | 143.9 (22.0) | 560 |  | 145.0 (22.6) | 531 | 1.1 (-1.6 – 3.7) | 3.6 (0.14 – 7.0) | 0.04 |
| 72h | 143.8 (20.3) | 548 |  | 142.5 (22.0) | 524 | -1.4 (-3.9 – 1.2) | 0.9 (-2.2 – 4.1) | 0.57 |
| 84h | 143.4 (21.5) | 499 |  | 144.0 (22.3) | 471 | 0.6 (-2.2 – 3.3) | 2.2 (-1.3 – 5.6) | 0.22 |
| 96h | 140.2 (19.5) | 489 |  | 142.6 (22.3) | 465 | 2.5 (-0.1 – 5.1) | 5.1 (1.8 – 8.3) | <0.01 |
| 108h | 142.4 (21.2) | 444 |  | 145.6 (21.5) | 416 | 3.2 (0.3 – 6.1) | 4.6 (1.0 – 8.2) | 0.01 |
| 120h | 140.6 (20.0) | 427 |  | 141.8 (20.8) | 418 | 1.2 (-1.6 – 4.0) | 3.1 (-0.7 – 6.8) | 0.11 |
| 132h | 139.9 (19.8) | 399 |  | 142.0 (21.1) | 381 | 2.1 (-0.7 – 5.0) | 1.8 (-1.9 – 5.4) | 0.34 |
| 144h | 138.6 (21.4) | 372 |  | 140.8 (21.3) | 367 | 2.2 (-0.9 – 5.3) | 3.3 (-0.6 – 7.2) | 0.10 |
| 156h | 140.3 (21.3) | 339 |  | 139.4 (21.4) | 345 | -0.9 (-4.1 – 2.3) | 1.3 (-2.9 – 5.5) | 0.56 |
| 168h | 136.6 (18.5) | 313 |  | 138.1 (19.8) | 316 | 1.4 (-1.5 – 4.4) | 2.9 (-1.1 – 6.7) | 0.15 |

Table 2a. Systolic blood pressure.

| **Time** | **Systolic blood pressure** | | | | | | | |
| --- | --- | --- | --- | --- | --- | --- | --- | --- |
|  | **No paracetamol** | |  | **Paracetamol** | |  | | |
|  | **Mean (SD)** | **N** |  | **Mean (SD)** | **N** | *DIM (95% CI)* | *Adjusted mean difference^#^* | *p-value* |
| 0 | 80.7 (16.8) | 723 |  | 80.5 (16.7) | 690 | -0.2 (-1.9 – 1.6) | - | - |
| 12h | 75.8 (15.6) | 693 |  | 75.3 (15.8) | 649 | -0.5 (-2.2 – 1.2) | -0.8 (-2.9 – 1.4) | 0.50 |
| 24h | 76.0 (14.3) | 674 |  | 74.2 (14.5) | 632 | -1.8 (-3.4 - -0.2) | -1.4 (-3.4 – 0.6) | 0.17 |
| 36h | 76.4 (14.9) | 644 |  | 75.7 (14.5) | 602 | -0.7 (-2.3 – 0.9) | -1.0 (-3.1 – 1.0) | 0.32 |
| 48h | 76.9 (14.5) | 620 |  | 75.4 (13.6) | 587 | -1.5 (-3.1 – 0.1) | -1.2 (-3.3 – 0.9) | 0.27 |
| 60h | 77.0 (13.6) | 560 |  | 76.7 (13.9) | 530 | -0.3 (-1.9 – 1.3) | 1.0 (-1.1 – 3.2) | 0.36 |
| 72h | 77.3 (13.5) | 548 |  | 76.0 (14.0) | 524 | -1.3 (-2.9 – 0.4) | 0.4 (-1.7 - 2.5) | 0.71 |
| 84h | 77.0 (13.6) | 499 |  | 77.2 (13.0) | 471 | 0.3 (-1.4 – 1.9) | 0.3 (-1.9 – 2.5) | 0.77 |
| 96h | 76.1 (13.4) | 489 |  | 76.9 (13.3) | 465 | 0.8 (-0.9 – 2.4) | 2.6 (0.4 – 4.9) | 0.02 |
| 108h | 76.8 (12.9) | 444 |  | 77.5 (13.5) | 416 | 0.7 (-1.1 – 2.5) | 1.6 (-0.7 – 4.0) | 0.18 |
| 120h | 76.8 (12.2) | 427 |  | 76.4 (13.1) | 418 | -0.4 (-2.1 – 1.3) | 2.0 (-0.2 – 4.3) | 0.08 |
| 132h | 76.0 (12.7) | 399 |  | 77.0 (12.3) | 381 | 1.0 (-0.7 – 2.8) | 0.9 (-1.4 – 3.2) | 0.44 |
| 144h | 76.2 (13.0) | 372 |  | 77.0 (13.5) | 367 | 0.9 (-1.0 – 2.8) | 1.3 (-1.2 – 3.7) | 0.31 |
| 156h | 75.4 (12.1) | 339 |  | 77.0 (12.0) | 345 | 1.6 (-0.2 – 3.4) | 2.2 (-0.2 – 4.6) | 0.08 |
| 168h | 75.9 (12.5) | 313 |  | 76.5 (12.1) | 316 | 0.6 (-1.3 – 2.6) | 1.6 (-0.9 – 4.2) | 0.20 |

Table 2b. Diastolic blood pressure

The mean systolic (Table 2a) and diastolic (Table 2b) blood pressure of patients randomised to paracetamol or to no paracetamol. Values depicted in mean (standard deviation). DIM: difference in means; h = hours; SD = standard deviation; 95% CI = 95% confidence interval. * = statistically significant. ^#^ Analyses were adjusted for age, sex, baseline blood pressure, stroke type, stroke severity, diabetes mellitus, hypertension, atrial fibrillation, pre-stroke modified Rankin Scale (mRS) score, baseline body temperature, country, time from stroke to trial treatment, allocation to the other treatment strata (metoclopramide, ceftriaxone), and treatment with intravenous thrombolysis or endovascular treatment.
