# Supplementary material for: The effects of prophylactic use of paracetamol on body temperature and blood pressure in elderly patients with acute stroke: Data from the PRECIOUS trial
Source: PLoS One. 2026 Feb 25;21(2):e0342937. doi: 10.1371/journal.pone.0342937 (PMC12935189; doi:10.1371/journal.pone.0342937)
Supplement: S2 Table — (DOCX) [file pone.0342937.s002.docx]

**Supplemental Table 1**. Mean heart rate in patients with and without paracetamol

| **Time** | **Heart rate** | | | | | | | |
| --- | --- | --- | --- | --- | --- | --- | --- | --- |
|  | **No paracetamol** | |  | **Paracetamol** | |  | | |
|  | **Mean (95% CI)** | **N** |  | **Mean (95% CI)** | **N** | *DIM (95% CI)* | *Adjusted difference* | *p-value* |
| 0 | 77.9 (76.6-79-2) | 719 |  | 78.0 (76.7-79.4) | 681 | 0.2 (-1.7 - 2.0) | - | - |
| 12h | 76.6 (75.3-77.8) | 684 |  | 74.3 (73.1-75.5) | 639 | -2.2 (-4.0 - -0.6) | -2.1 (-4.3 - -1.2) | <0.01* |
| 24h | 77.5 (76.2-78.7) | 668 |  | 74.8 (73.6-76.0) | 623 | -2.6 (-4.4 - -0.9) | -1.2 (-3.4 - 1.0) | 0.05 |
| 36h | 76.7 (75.5-78.0) | 633 |  | 74.3 (73.1-75.6) | 598 | -2.4 (-4.2 - -0.6) | -1.5 (-3.7 - 0.8) | 0.27 |
| 48h | 76.6 (75.4-77.8) | 610 |  | 75.1 (73.7-76.4) | 582 | -1.6 (-3.4 - 0.3) | -1.2 (-3.5 - 1.1) | 0.20 |
| 60h | 77.1 (75.8-78.5) | 550 |  | 74.7 (73.3-76.0) | 525 | -2.5 (-4.4 - -0.5) | -0.5 (-2.8 - 1.7) | 0.30 |
| 72h | 76.2 (74.9-77.6) | 538 |  | 74.2 (72.9-75.6) | 519 | -1.5 (-3.4 - 0.4) | -0.5 (-2.8 - 1.7) | 0.65 |
| 84h | 75.2 (73.8-76.5) | 488 |  | 74.2 (72.9-75.6) | 469 | -1.0 (-2.9 - 0.9) | 0.0 (-2.4 - 2.4) | 0.65 |
| 96h | 76.3 (74.8-77.7) | 483 |  | 74.9 (73.5-76.4) | 462 | -1.3 (-3.4 - 0.7) | -0.9 (-3.4 - 1.6) | 0.98 |
| 108h | 76.3 (74.8-77.7) | 433 |  | 74.9 (73.4-76.5) | 410 | -1.3 (-3.4 - 0.7) | 1.1 (-1.5 - 3.8) | 0.50 |
| 120h | 75.7 (74.3-77.0) | 417 |  | 75.8 (74.2-77.4) | 411 | 0.1 (-2.0 - 2.2) | 1.1 (-1.5 - 3.8) | 0.41 |
| 132h | 75.8 (74.3-77.4) | 387 |  | 74.9 (73.4-76.4) | 372 | -0.9 (-3.1 - 1.2) | -0.9 (-3.5 - 1.7) | 0.51 |
| 144h | 74.7 (73.2-76.3) | 362 |  | 75.8 (74.1-77.4) | 363 | 1.0 (-1.2 - 3.2) | 1.7 (-11 - 4.5) | 0.25 |
| 156h | 74.6 (72.9-76.2) | 327 |  | 76.0 (74.3-77.7) | 334 | 1.5 (-0.9 - 3.8) | 0.2 (-2.5 - 3.0) | 0.88 |
| 168h | 75.4 (73.8-77.0) | 305 |  | 75.4 (73.8-77.1) | 308 | 0.0 (-2.3 - 2.3) | 2.8 (0.2 - 5.4) | 0.03* |

The mean heart rate of patients randomized to paracetamol or to no paracetamol. Values depicted in mean (95% confidence interval). DIM: difference in means; h = hours; 95% CI = 95% confidence interval. * = statistically significant.
