# Supplementary material for: The effects of prophylactic use of paracetamol on body temperature and blood pressure in elderly patients with acute stroke: Data from the PRECIOUS trial
Source: PLoS One. 2026 Feb 25;21(2):e0342937. doi: 10.1371/journal.pone.0342937 (PMC12935189; doi:10.1371/journal.pone.0342937)
Supplement: Attachment — Submitted filename: Response to Reviewers 200825.docx [file pone.0342937.s004.docx]

Dear Dr. Yusuff,

Thank you for considering the publication of our paper in your journal and for the constructive criticism of the reviewers. We have read the suggestions for improvement with detail and adjusted the manuscript where appropriate. Please see below our response to each point raised by the academic editor and reviewers.

Journal Requirements:

When submitting your revision, we need you to address these additional requirements.

1. Please ensure that your manuscript meets PLOS ONE's style requirements, including those for file naming. The PLOS ONE style templates can be found at

<https://journals.plos.org/plosone/s/file?id=wjVg/PLOSOne_formatting_sample_main_body.pdf> and

[https://journals.plos.org/plosone/s/file?id=ba62/PLOSOne_formatting_sample_title_authors_affiliations.pdf](https://track.editorialmanager.com/CL0/https:%2F%2Fjournals.plos.org%2Fplosone%2Fs%2Ffile%3Fid=ba62%2FPLOSOne_formatting_sample_title_authors_affiliations.pdf/1/010f01979cfaf372-f7fa630a-1e92-4370-84f0-ae3479677e57-000000/xSlhRYbGS7ljgHHdlTc-Pty_cB8GtferVIkQRSzfcnw=216).

The paper has been adjusted according to the above-mentioned style requirements.

2.  We note that the grant information you provided in the ‘Funding Information’ and ‘Financial Disclosure’ sections do not match.

When you resubmit, please ensure that you provide the correct grant numbers for the awards you received for your study in the ‘Funding Information’ section.

In the ´Funding Information´ section, it is not possible to select one of the pre-defined options that exactly resembles our standard funding statement: ´This project has received funding from the European Union´s Horizon 2020 research and innovation programme under grant agreement No: 634809. Currently, we have selected the heading ´H2020 European Research Council´, as this option best resembles the funding statement.

3. Thank you for stating the following financial disclosure:

 [PRECIOUS was funded by the European Union’s Horizon 2020 research and innovation programme (634809).].

Please state what role the funders took in the study.  If the funders had no role, please state: ""The funders had no role in study design, data collection and analysis, decision to publish, or preparation of the manuscript.""

If this statement is not correct you must amend it as needed.

Please include this amended Role of Funder statement in your cover letter; we will change the online submission form on your behalf.

The statement has been added to the revised cover letter.

4. In the online submission form, you indicated that [De-identified individual participant data and a data dictionary defining each field in the set can be made available to others upon reasonable request to the corresponding author, subject to privacy regulation.].

All PLOS journals now require all data underlying the findings described in their manuscript to be freely available to other researchers, either 1. In a public repository, 2. Within the manuscript itself, or 3. Uploaded as supplementary information.

This policy applies to all data except where public deposition would breach compliance with the protocol approved by your research ethics board. If your data cannot be made publicly available for ethical or legal reasons (e.g., public availability would compromise patient privacy), please explain your reasons on resubmission and your exemption request will be escalated for approval.

The statement has been adjusted. Unfortunately, we cannot make data publicly available because of strict privacy regulations and the fact that patients did not explicitly provide consent for public disclosure of their coded data. In the informed consent form, that was signed by the participants and approved by the central ethical committee, it was not mentioned that the data could be made publicly available. We also discussed the situation with the privacy officer of the UMC Utrecht and he agrees with the statement above.

The email address that can be used for future references is the general email address of the Scientific Bureau of the Neurology Trial Department at UMC: [**trialbur@umcutrecht.nl**](mailto:trialbur@umcutrecht.nl).

5.  Please include captions for your Supporting Information files at the end of your manuscript, and update any in-text citations to match accordingly. Please see our Supporting Information guidelines for more information: <http://journals.plos.org/plosone/s/supporting-information>.

The captions for the Supporting Information files have been added to the end of our manuscript.

6. Thank you for stating the following in the Competing Interests section:

[JCdJ, WMS, HR report grants from the European Union, all paid to their institution. PMB is Stroke Association Professor of Stroke Medicine and an emeritus NIHR Senior Investigator; he has received grants from the NIHR and British Heart Foundation, and funding for consultancy from CoMind and DiaMedica. DvdB reports having received research grants from the European Union, The Netherlands for Health Research and Development, ItsMe Foundation, AMC Foundation and Roche; none related. AHA reports research grants from Boehringer Ingelheim, lectures fee from Abbvie, BMS/Pfizer, Novartis, Roche and Teva and participation in Advisory Board for Lundbeck, Abbvie and MSD; none related. AC reports grants from the European Union and Lombardy region, for research paid to his institution, and fees as consultant or lecturer from Alexxion Pharma, Daiichi Sanky, and Italfarmaco. JK reports lecturer fees from Boehringer Ingelheim, Pfizer and Servier, and travel grants from Boehringer Ingelheim and Servier, none related. MDC has received research grants from the Werner Otto Foundation, speakers honoraria from AstraZeneca and was funded by the Faculty of Medicine, University of Kiel, Germany through its Advanced Clinician Scientist Programme, all outside the submitted work. IKJ reports lectures and travel grants from Merck, Novartis and Roche, Angelini Pharma and travel grants from Boehringer Ingelheim, none related. GT report grants from the European Union, German Research Foundation, German Federal Ministry of Education and Research, German Innovation Fund for research paid to his institution, and fees as consultant or lecturer from Acandis, Alexion, Amarin, Bayer, Boehringer Ingelheim, Daiichi Sanky, BristolMyersSqibb/Pfizer, and Stryker. HBvdW reports having received grants from the European Union, the Dutch Heart Foundation, and Stryker for research, and funding for consultancy from Bayer, Boehringer Ingelheim, and TargED, all paid to his institution.].

We note that you received funding from a commercial source: [European Union, NIHR, British Heart Foundation, Abbvie, BMS/Pfizer, Lombardy region, Alexxion Pharma, Daiichi Sanky, Italfarmaco, Boehringer Ingelheim, Pfizer and Servier, Werner Otto Foundation, Faculty of Medicine, University of Kiel, Merck, Novartis and Roche, Angelini Pharma, German Research Foundation, German Federal Ministry of Education and Research, Acandis, Alexion, Amarin, Bayer, Boehringer Ingelheim, Daiichi Sanky, BristolMyersSqibb/Pfizer, and Stryker]

Please provide an amended Competing Interests Statement that explicitly states this commercial funder, along with any other relevant declarations relating to employment, consultancy, patents, products in development, marketed products, etc.

Within this Competing Interests Statement, please confirm that this does not alter your adherence to all PLOS ONE policies on sharing data and materials by including the following statement: ""This does not alter our adherence to PLOS ONE policies on sharing data and materials.” (as detailed online in our guide for authors <http://journals.plos.org/plosone/s/competing-interests>).  If there are restrictions on sharing of data and/or materials, please state these. Please note that we cannot proceed with consideration of your article until this information has been declared.

Please include your amended Competing Interests Statement within your cover letter. We will change the online submission form on your behalf.

The amended Competing Interests Statement has been added to our cover letter. Of note, the study itself was only funded by the European Union, which is non-commercial. Individual investigators did have their own competing interests, that were noted in this section.

7. One of the noted authors is a group [PRECIOUS investigators]. In addition to naming the author group, please list the individual authors and affiliations within this group in the acknowledgments section of your manuscript. Please also indicate clearly a lead author for this group along with a contact email address.

Given the length of the list of all the PRECIOUS investigators, the individual authors have been added as supplemental information (S3 appendix).

[Note: HTML markup is below. Please do not edit.]

Reviewers' comments:

Reviewer's Responses to Questions

**Comments to the Author**

1. Is the manuscript technically sound, and do the data support the conclusions?

The manuscript must describe a technically sound piece of scientific research with data that supports the conclusions. Experiments must have been conducted rigorously, with appropriate controls, replication, and sample sizes. The conclusions must be drawn appropriately based on the data presented.

Reviewer #1: Partly

Reviewer #2: Partly

Reviewer #3: Yes

2. Has the statistical analysis been performed appropriately and rigorously?

Reviewer #1: No

Reviewer #2: Yes

Reviewer #3: Yes

3. Have the authors made all data underlying the findings in their manuscript fully available?

The [PLOS Data policy](http://track.editorialmanager.com/CL0/http:%2F%2Fwww.plosone.org%2Fstatic%2Fpolicies.action%23sharing/1/010f01979cfaf372-f7fa630a-1e92-4370-84f0-ae3479677e57-000000/0IokbgjO2A5leHeYTzT0pycNCOMyNcupSMUVtTMXKAo=216) requires authors to make all data underlying the findings described in their manuscript fully available without restriction, with rare exception (please refer to the Data Availability Statement in the manuscript PDF file). The data should be provided as part of the manuscript or its supporting information, or deposited to a public repository. For example, in addition to summary statistics, the data points behind means, medians and variance measures should be available. If there are restrictions on publicly sharing data—e.g. participant privacy or use of data from a third party—those must be specified.

Reviewer #1: Yes

Reviewer #2: Yes

Reviewer #3: Yes

4. Is the manuscript presented in an intelligible fashion and written in standard English?

PLOS ONE does not copyedit accepted manuscripts, so the language in submitted articles must be clear, correct, and unambiguous. Any typographical or grammatical errors should be corrected at revision, so please note any specific errors here.

Reviewer #1: Yes

Reviewer #2: Yes

Reviewer #3: Yes

5. Review Comments to the Author

Reviewer #1: Reviewer Comments
This manuscript evaluates the effects of prophylactic paracetamol administration on body temperature and blood pressure among elderly acute stroke patients, leveraging data from the international PRECIOUS randomized controlled trial (RCT). The analysis of 1,419 patients aimed to validate previous findings from studies such as the PAIS trial. Your primary conclusion—that paracetamol modestly reduces mean body temperature and significantly decreases the occurrence of subfebrile or febrile episodes without affecting blood pressure—is clinically relevant and clearly articulated.

Overall, the manuscript is logically structured and well-presented. The international, multicenter RCT design ensures robust statistical power and enhances external validity. However, important concerns regarding the accuracy and transparency of your statistical analysis—specifically related to the chi-square test results—need to be urgently addressed, as these issues directly influence the credibility and interpretability of your findings.

Specific Comments:
1. Multiple Comparisons and Potential for Inflated Type I Error:
Given that statistical analyses were conducted across multiple time points (every 12 hours over a 7-day period), there is a significant risk of inflated Type I errors (false positives). The manuscript does not clearly indicate whether any statistical corrections (such as Bonferroni, Holm, or False Discovery Rate adjustments) for multiple comparisons were applied.

Recommendation:
Explicitly clarify whether any correction for multiple comparisons was performed. If corrections were not applied, please provide a clear justification for this decision in the manuscript. Transparently acknowledging this issue will considerably enhance the methodological rigor and reliability of your analysis.

Response:

No corrections for multiple comparisons were applied, as the analyses were considered exploratory and hypothesis-generating rather than confirmatory hypothesis-testing. Furthermore, the multiple measurements were not independent, as the measurements were repeated within the same participants. These two justifications have now been included in the Methods section of the paper. We discussed this with two co-authors of the paper who are also statistician, and they agreed with the statement above.

2. Clarification Regarding "Adjusted Difference" in Table 2:
In Table 2, you report an 'Adjusted difference'; however, the methods section lacks a clear explanation of how these adjusted mean differences were derived.

Recommendation:
Clearly explain the statistical approach and the specific covariates used for adjustment when calculating these differences. This should be explicitly detailed in the statistical methods section of your manuscript to ensure clarity and reproducibility.

Response:

The statistical analyses performed to obtain the results of Table 2 were carried out according to the ´statistical analysis´ paragraph in the ´Methods´ section of the paper. We performed a multiple linear regression and adjusted for the covariates defined in the statistical analysis paragraph: analyses were adjusted for age, sex, baseline body temperature, stroke type, stroke severity, diabetes mellitus, hypertension, atrial fibrillation, pre-stroke modified Rankin Scale (mRS) score, baseline body temperature, country, time from stroke to trial treatment, allocation to the other treatment strata (metoclopramide, ceftriaxone), and treatment with intravenous thrombolysis or endovascular treatment.

The specific covariates have been added to the legend of the Table.

3. Major Concern – Discrepancies in Chi-square Test Results (Table 3):
In your methods section, you indicated that differences in the rate of subfebrile temperatures or fever were assessed using chi-square tests, with results presented in Table 3. Upon independently recalculating these chi-square values using the provided data, I identified discrepancies between your reported p-values and the independently calculated ones. While these discrepancies generally do not appear large enough to substantially alter your primary conclusions, they nevertheless undermine the robustness and credibility of the statistical analysis performed.

Notably, these differences remained even when employing Fisher’s Exact Test, suggesting a systemic issue rather than simply the choice of statistical test. Furthermore, because the variables utilized in your multiple regression analyses were not fully provided, it was impossible for me to independently validate those analyses as well.

Recommendation:
Please urgently re-examine and confirm the accuracy of all chi-square analyses presented in Table 3 using reliable statistical software (e.g., R, Python, SPSS, Stata). Provide transparent recalculations of these analyses along with clearly documented steps or scripts to facilitate external verification. Clarifying this issue is critical to ensure confidence in the reported results and their interpretation. I have shared with the editorial team the Python script and resulting calculations I performed for further reference and verification.

Addressing these concerns will significantly enhance the methodological clarity, transparency, and overall quality of your manuscript.

Response:

The chi-square analyses of Table 3 have been repeated using the same software (SPSS), and the results are identical to the current numbers in Table 3. In the revision, a document containing the exact SPSS output (2x2 contingency tables & chi-square analyses) of each calculation has been added.

It seems that the reviewer applied Yates correction to the chi-square test. We did not use Yates´ correction (or Fisher exact test), because the sample was of considerable size and we felt that Yates´ correction was not appropriate. After discussion with our statistician, we decided not to add this correction.

Reviewer #2: This was a substudy of the PRECIOUS study. Prophylactic administration of paracetamol (acetaminophen) has been reported to reduce body temperature in the first days after stroke and to reduce blood pressure on the first day. The investigators wanted to validate these findings in the randomized PRECIOUS trial. The paper was well presented from the statistical perspective.

Absolute mean differences (with 95% confidence intervals (CI)) in body temperature, heart rate, and systolic and diastolic blood pressure between patients randomized to paracetamol versus no paracetamol, were assessed by multiple linear regression for each 12-hour time point after randomization in the first 7 days after randomization. Performing this type of timepoint analysis the investigators concluded that prophylactic use of paracetamol resulted in a modest reduction in mean body temperature and almost halved the rate of subfebrile temperatures or fever at 24 hours after stroke, but had no effect on blood pressure.

Figures 1a to 1c basically demonstrated the results. The analysis was by time and obviously was very simple. Although this was not the intent of the original trial and the results are mostly observational, one wonders why the investigators did not attempt a repeated measures type of analysis with treatment and time as the factors and getting a p-value for these overall effects for the course of this short study.

Response:

We chose to perform separate logistic regressions for each time point rather than using a repeated measures approach (e.g. linear mixed model), because our primary interest was to assess the effect of the medication at each individual time point rather than estimate an overall longitudinal effect. We acknowledge that this method does not account for within-subject correlation, which is a limitation. We discussed this with two co-authors of the paper who are also statistician, and they agreed with the statement above.

Also please comment on the interaction noticed in the plots, mostly at the end of the Figures 1a and 1b.

Response:

The interactions between time and treatment in the plots (Figure 1a en Figure 1b) are a reflection that paracetamol does have a modest effect on mean body temperature. In the first 96 hours, there is a numerical (and frequent statistical) difference in body temperature between patients randomised to paracetamol en no paracetamol. After 96 hours, when paracetamol prophylaxis stops, mean body temperature of both groups become similar. We feel that this is sufficiently described in the ´Results´ section of the paper.

Also since a multiple regression analysis adjustment was made, was there any attempt to examine the significance of the baseline values on the outcome?

No analyses were performed to assess the significance of baseline values on the outcome.

.

Reviewer #3: Subject: Peer Review Feedback – Manuscript ID: PONE-D-25-22053

Title: The effects of paracetamol on body temperature and blood pressure in patients with acute stroke. Data from the PRECIOUS trial

Thank you for the opportunity to review this manuscript. The submitted work represents a secondary analysis of the PRECIOUS trial - an international, multicenter, 3×2 factorial, randomized, controlled trial investigating the effects of paracetamol, metoclopramide, and ceftriaxone in older adults (≥66 years) with acute stroke and NIHSS score ≥6. The PRECIOUS trial’s main outcomes, protocol, and statistical analysis plan have previously been published in peer-reviewed journals.

This manuscript investigates a clinically relevant topic – i.e. the effects of paracetamol on temperature and blood pressure (BP) in acute stroke patients. While the study addresses an important area with limited existing evidence, the manuscript in its current form requires significant revision to meet the standards of PLOS ONE. Below are my detailed comments and suggestions for improvement.

General Comments
• The manuscript is generally well-structured and presents interesting findings. However, improvements are needed in language, grammar, and scientific writing quality.

The paper has been carefully revised and has been edited in several places to enhance the quality of the language, grammar and writing.

• I strongly recommend a thorough proofreading by a native English speaker with experience in clinical research to enhance clarity and coherence.

The paper has been re-reviewed by the three authors who are native English speakers and has been edited where appropriate.
• The rationale for conducting this secondary analysis is weakly presented. A more robust literature review is needed to clearly articulate the knowledge gap this study addresses.

The ´introduction´section has been expanded and extra relevant literature is added. Based on limitations of the previous literature on this topic, we have emphasized the added value of our current study.

• All abbreviations should be defined at first mention and used consistently throughout. For example, NIHSS was not defined in the abstract.

We reviewed the paper and all abbreviations are now defined at the moment the abbreviation is first mentioned.
• Subheadings in various sections are inconsistently formatted.
The style of subheadings is adjusted.

Title
• The current title does not fully reflect the study population. Since all included participants were ≥66 years, I recommend revising the title to include “older adult patients” for accuracy.

We have added the word ´elderly´ to the title to more accurately reflect the study population.

Abstract
• Background and Aims: The rationale for the study is not clearly stated. Prior studies have reported on paracetamol’s effects in stroke, what new aspect is being investigated here?

In the abstract, we state our aim to validate previous findings in the PRECIOUS trial. We have now added that this study examines the effect of paracetamol on body temperature over a longer period than has been investigated previously.
• Methods: The outcome descriptions must be consistent with the main text (e.g., diastolic BP is included in the main manuscript but not in the abstract).

Diastolic blood pressure has been added as a primary outcome to abstract, in accordance to the methods section of the paper.
• Results: When referring to “blood pressure,” clarify whether this means “systolic blood pressure” specifically.

We have added that paracetamol did not reduce systolic or diastolic blood pressure.

Introduction
• The introduction should better define what is known, what remains unknown, and the research gap.

In line with a previous answer, we have emphasized the added value of our current study.
• Line 72: Specify what is meant by “functional outcomes.”

By ´functional outcomes´, we refer to outcomes as defined by modified Rankin Scale (mRS). We have chosen not to change the original phrasing, as ´functional outcome´ is common terminology in stroke literature and understood within the field.
• Line 77: Define what is considered a “high dose” of paracetamol and cite relevant sources.

The dose of paracetamol used in the PAIS trial has been added to this part of the introduction. A reference to the source is present at the end of the sentence.
• Line 83–84: Expand on the association between elevated temperature/BP and poor clinical outcomes, including which outcomes.

We have changed ´poorer outcome´ to ´recurrence of cardiovascular events, poor functional outcome and mortality´. These outcomes were the primary outcomes in the cited studies.
• Line 87: Replace “by contrast” with “in contrast.”

The wording has been corrected.
• Line 90–92: Clarify the objective statement, which is currently confusing due to the introduction of PAIS.

We have rephrased the objective of the study.

Methods
• Lines 96–104: Please cite the original publication(s) of the PRECIOUS trial. The long sentence here should be broken into shorter, clearer ones.

The PRECIOUS trial has been cited at the beginning of the paragraph. The long sentence has been broken into multiple shorter sentences.
• Line 109–110: Dates should follow academic and journal-specific formatting.

The dates are now displayed correctly.
• Line 113: Ensure uniformity in formatting of subheadings (some are bold, others italicized).

The subheadings have been adjusted in accordance with the journal’s formatting guidelines.

• Line 119–122: Clarify the statement about omitting one randomization stratum.

We have changed to word ´omit´ to ´censor´. In addition, we have added an example to clarify the concept of censoring.
• Line 136–137: Ensure consistency in the list of primary outcomes across sections.

The ´diastolic blood pressure´ has been added to the abstract as primary outcome. The abstract, methods, results and discussion section now match regarding the primary outcome.
• Line 151: Avoid redundancy - baseline body temperature was already listed.

Baseline body temperature was indeed mentioned twice. This has been changed.
• Line 155–156: “Statistical significance was set at 95%” is incorrect. Please revise to “a p-value < 0.05 was considered statistically significant.”

The sentence has been changed to the suggested alternative.
• Line 156: The city of manufacture for SPSS should be verified. Chicago, IL may not be accurate.

Indeed, the headquarters of SPSS was located in Chicago, IL. However, IBM has fully acquired SPSS Inc. in 2010. The headquarters of IBM is located in Armonk, NY.

Results
• Line 177 onwards (last paragraph) – I see potential reporting bias in the paper. The reporting of systolic BP findings is detailed, but diastolic BP is completely omitted, despite being a listed primary outcome.

In the first sentence of the paragraph, we now emphasize that paracetamol does not lower systolic or diastolic blood pressure at any time point. In the following sentences, we explain that the prophylactic use of paracetamol was associated with an increase of systolic blood pressure on 2 time points and with an increase of diastolic blood pressure on 1 time point. All data regarding systolic and diastolic blood pressure are illustrated in supplemental table 1.
• Please present findings on diastolic BP, in the text and/or through an additional table or figure.

The text has been updated. Figure 1C already illustrates diastolic blood pressure, and supplemental Table 1 has been added to provide a complete overview of all blood pressure values.

Discussion
• The discussion is too descriptive. A more critical interpretation of the findings in the context of existing literature is required. Avoid redundancy and focus on key outcomes and their implications.
The discussion has been modified on several points based on the reviewers´ comments. In the second to sixth paragraph of the discussion, our results are carefully compared to previous results in the literature. We explore potential reasons for any discrepancies. We feel that the current version of the discussion forms a critical interpretation of our findings.

• Line 188: Reword “by a very modest 0.1°C” to improve clarity and tone.

We have rephrased the sentence.
• Line 191: Please cite the previous randomized trial referenced.

The citation has been added.
• Line 193–194 and Line 204–205: Address grammatical errors in these sentences.

In line 193-194, we have corrected ´as´ into and´. Line ´204-205´ has been rewritten.
• Consider including interpretation of findings related to DBP and HR, which are currently underrepresented.

The discussion included an extended paragraph on blood pressure. Given that a previous study found an effect on systolic blood pressure, this aspect is explored in greater detail in the paragraph. Diastolic blood pressure is now explicitly mentioned, where appropriate. In contrast, because paracetamol had no effect on heart rate, this association is only briefly addressed.

Conclusion
• The conclusion should be more reflective of the study’s findings and emphasize clinical implications. Ensure alignment with the study’s objectives.
The paragraph has been rewritten to better reflect the study´s findings.

Tables
• Table 1:
o Title of Table 1 is not descriptive enough.

The title has been changed.

o Is the age presented as mean (SD), please specify in the first column?

The data is indeed presented as mean with standard deviation. The legend of the table has been adjusted.
o Medical history: Some have % in the first column, others like AF and DM do not have the symbol of %.

Indeed, this is inconsistent. We have changed the table accordingly.
• Table 2:
o Expand the title for clarity – it is currently not descriptive enough.

The title has been expanded for clarity. The title of table 3 has been adapted as well.
o A similar table for diastolic BP should be created and included.
A similar table for systolic and diastolic blood pressure has been added as supplemental table (supplemental table 1).

References
• Ensure all in-text citations and reference formatting follow PLOS ONE’s guidelines.
The references has been revised according to the journal´s formatting guidelines.

Reviewer’s Summary
In summary, the manuscript has the potential to contribute valuable data on a topic of interest in stroke management. However, substantial revision is necessary to clarify its rationale, improve its consistency, and enhance scientific rigor. I hope the authors will find these comments constructive in revising the manuscript.

**Do you want your identity to be public for this peer review?** For information about this choice, including consent withdrawal, please see.

Reviewer #1: No

Reviewer #2: No

Reviewer #3: No
