# Supplementary material for: The effects of prophylactic use of paracetamol on body temperature and blood pressure in elderly patients with acute stroke: Data from the PRECIOUS trial
Source: PLoS One. 2026 Feb 25;21(2):e0342937. doi: 10.1371/journal.pone.0342937 (PMC12935189; doi:10.1371/journal.pone.0342937)
Supplement: Attachment — Submitted filename: Response to Reviewers 200126.docx [file pone.0342937.s005.docx]

Dear Dr. Yusuff,

Thank you for considering the publication of our paper in your journal and for the constructive criticism of the reviewers. We have read the suggestions for improvement with detail and adjusted the manuscript where appropriate. Please see below our response to each point raised by the academic editor and reviewers.

---

Reviewers' comments:

6. Review Comments to the Author

Please use the space provided to explain your answers to the questions above. You may also include additional comments for the author, including concerns about dual publication, research ethics, or publication ethics. (Please upload your review as an attachment if it exceeds 20,000 characters)

Reviewer #2: (No Response)

Reviewer #3: 1. I suggest the title should include the word "prophylactic" as the management here was not therapeutic. I suggest something like: "The Effects of Prophylactic Paracetamol use on Body Temperature and Blood Pressure in Patients with Acute Stroke".
2. I still feel Figures 1a, 1b, 1c are not of good resolution. Kindly improve the resolution.

The title has been adapted accordingly.

The resolution of Figure 1B deteriorated during the transfer from Excel to Word. The figure has now been recopied while preserving the original resolution. The resolution of the figures in the Word files appears sufficient at this point. The Word files were subsequently converted to TIFF using an alternative method, resulting in significantly improved image quality. Off note, the quality of the images is low in the overall PDF file of the submission, but if the images are downloaded in the PDF file, the quality is high.
